# Becoming a secondary actor of one's own life: A qualitative study of the experiences of informal caregivers in the care of people with chronic pain

**DOI:** 10.1111/hex.13671

**Published:** 2022-12-08

**Authors:** Helena De Sola, Inmaculada Failde, Itziar Estalella, Amaia Maquibar

**Affiliations:** ^1^ The Observatory of Pain University of Cádiz Cádiz Spain; ^2^ Biomedical Research and Innovation Institute of Cádiz (INiBICA) Research Unit Puerta del Mar University Hospital, University of Cádiz Cádiz Spain; ^3^ Preventive Medicine and Public Health Area University of Cádiz Cádiz Spain; ^4^ Department of Nursing I, Faculty of Medicine and Nursing University of the Basque Country UPV/EHU Bizkaia Leioa Spain

**Keywords:** burden, chronic pain, experiences, informal caregivers

## Abstract

**Introduction:**

The physical limitations experienced by people with chronic pain (CP) produce a greater need for care and assistance, most of which is provided by an informal caregiver (IC). Despite the key role ICs play in the everyday lives of individuals living with CP, knowledge about their experiences and needs is limited. We aimed to address this limitation by exploring the experiences of IC of people with CP.

**Methods:**

This is a qualitative descriptive study using semistructured interviews. Participants were 12 ICs purposively chosen from the Unit of Pain at the University Hospital in Cádiz. Individual interviews were recorded, transcribed verbatim and analysed following thematic analysis.

**Results:**

We developed one overarching theme ‘Becoming a secondary actor of one's own life’ and three themes: 1. Key elements that shape a caregiver's experiences; 2. It's the hand that life dealt me; 3. The burden of being a caregiver and coping strategies.

**Conclusions:**

This study's findings highlight how the CP impacts IC lives. Being an IC for a relative with CP became the most important role in the IC's life, to the point of casting a shadow over their own needs. Besides, participants felt not having other options but to keep going with that role. Yet, the context was essential in shaping the experiences as caregivers and the burden derived from caregiving. In this line, differences related to gender roles were found in the narratives of participant women and men.

**Patient or Public Contribution:**

Participants were purposively chosen from the Unit of Pain at the University Hospital ‘Puerta del Mar’ who attended the consultation accompanying their relatives. All the eligible participants were approached by the clinician. After this initial approach by the clinician, one of the researchers met the potential participant and they went to a quieter place in a clinical setting for the interview, before which the participant was shown a letter with more comprehensive information about the study and its aim. The participants were left alone to read and think carefully before giving their written informed consent. Participation was voluntary and the subjects received no financial contribution for their time.

## 1 INTRODUCTION

Chronic pain (CP) is an important public health problem that affects between 10% and 30% of the adult population in Europe, and 17% of the general Spanish population.^1^ Additionally, this prevalence is expected to increase in coming years, due to the ageing of the global population and prolonged exposure to risk factors such as obesity or occupational factors.^2^, ^3^, ^4^ The mean duration of CP in Spain is 10 years,^5^ with the majority of the sufferers reporting moderate or severe pain.^5^, ^6^, ^7^ The physical limitations experienced by people with CP produce a greater need for care and assistance, most of which is provided by an informal caregiver (IC), that is, an unpaid family member or friend who provides assistance with everyday activities.^8^ In this vein, the IC makes an important contribution to the formal health system. The mean length of CP mentioned above, suggests that the ICs have to accompany their relative for a prolonged period of time.

The role played by ICs is not unique to CP patients, as they play an essential role in many diseases. Therefore, the needs, experiences and consequences of being an IC have been widely studied, especially in the case of IC of the elderly, cancer patients, mental health and in palliative care. In the case of ICs for cancer patients, a study found that the new role and tasks performed can be emotionally, physically, socially and financially demanding.^9^ Similarly, ICs of individuals with mental illness were found to have limited social and recreational activities as a result of their dedication to the patient.^10^ Additionally, financial stress, derived from being responsible for continuous care and maintaining the family income, was identified as one of the most crucial challenges in daily living, contributing to mental illnesses among ICs.^11^, ^12^

More limited knowledge is available about the experiences of ICs of individuals living with CP patients.^13^ Studies have found there are some commonalities with IC of other diseases and some specificities related to the characteristics of CP. Similarly to what happens in other diseases, the IC has to perform new tasks related to the role, such as administering medications, dealing with the possible side effects of treatment and managing medical appointments. Some studies have shown^14^, ^15^ how ICs share the emotional experiences of people in pain, including stress, distress and insomnia which significantly reduces their own well‐being. Being the everyday ‘witness to pain’ obliges IC to act as the reporter and defender of the pain to those who may question the sincerity of the pain^13^ due to its ‘invisibility’,^16^ for example by explaining to the physician the characteristics of the pain of their family member.^13^ The heterogeneity in individual's pain perceptions, and the biological, psychological and social nature of pain, requires not only a clinical approach but also fulfilling the social expectancies and responsibilities to offer informal care.^13^ Additionally, CP is frequently associated with other pathologies, making the role of the IC more challenging and complex.

Experiences of IC have been found, as with other diseases, to vary in relation to sex and gender. Due to traditional gender roles, women, who are the vast majority of IC,^17^ frequently assume the greatest share of responsibilities for maintaining the family's organization and providing nurturance to family members.^17^ This gender gap is especially visible in mature‐age caregivers, that is, those between 40 and 64 years,^18^ who have been described in previous studies as the ‘sandwich generation’.^19^ These women find themselves in a situation of caring for elderly parents, having dependent children and remaining active in the labour market.^8^ Consequently, women may experience losses of identity, privacy and time for themselves and are at greater risk of poorer health than their male counterparts.^20^

Taking into account that CP prevalence is already significant and is expected to increase in coming years,^4^ leading to an increase in the number of ICs for individuals living with CP, and that previous studies have found some specificities related to the characteristics of CP, this study aimed to explore the experiences of ICs of people with CP to better understand and respond to their needs. Likewise, these experiences were analysed from a gender perspective.

## 2 METHODS

### 2.1 Study design

This is a qualitative descriptive study^21^ in which data were collected through semistructured interviews to explore the experiences of ICs of CP patients.

### 2.2 Setting and participants

The participants were the main IC of a patient with CP, understanding IC as the person responsible for the help needed by the patient to perform basic daily activities during the main part of the day without receiving a salary for providing this help.

The inclusion criteria for the participants were adults who accompanied their relative with CP to the consultation of the Pain Clinic of the Puerta del Mar University Hospital and consider themselves to be him/her main caregiver. After an analysis of the medical record and a physical evaluation of the CP patient, the clinician asked the person who accompanied him or her if they were the main IC of the CP patient. In case him/her responded affirmatively, the clinician explained to them the aim of the study. All the potential participants answered affirmatively to the question and agreed to participate. After this initial approach by the clinician, the interviewer met the participant and they went to a quieter place in a clinical setting for the interview.

### 2.3 Data collection

Data collection took place between May and October 2021. Twelve ICs agreed to participate in the interviews (Table 1), which were conducted by HDS. Individual, semistructured, qualitative interviews following a guide were conducted in Spanish. The guide was based on open‐ended questions developed with guidance from the literature regarding CP and IC experiences (Table 2). Interviews were audio‐recorded, transcribed verbatim and anonymized. All names used here are pseudonyms. We conducted interviews until very similar experiences were described in the last interviews as the previous interviews.

**Table 1:** Characteristics of the sample

	Gender	Age	Income/occupation	Relationship	Time dedicated to care	Task performed
Natalia	Female	44	Total permanent disability	Wife	Since 2016	24 h assistance
Juan	Male	72	Retired (bricklayer)	Husband	Since 2012	24 h assistance
Marco	Male	77	Total permanent disability (carpenter)	Husband	Since 2017	24 h assistance
Pablo	Male	73	Retired (Waiter)	Husband	‐	Shopping and accompany to doctor
Rocío	Female	34	Teacher	Wife	Since 2019	Housework and tasks involving carrying weight
Celia	Female	46	Administrative assistant	Daughter	Since 2020	Company and supervision of all mother's activities
Marta	Female	47	Unemployed	Daughter	Since 2012	Housework and company
Elena	Female	51	Teacher	Daughter	Since 2016	Company and supervision
Ana	Female	70	Unemployed	Wife	‘a lot of years’	Housework, company and supervision
Javier	Male	68	Retired (Sailor)	Partner	Since 2015	Some housework, company and supervision
Miriam	Female	66	Businesswoman	Wife	Since 2010	24 h assistance
Milagros	Female	45	Medical assistant	Daughter	Since 2013	Company and Supervision

**Table 2:** Interview guide used for the semistructured interviews

B1. Sociodemographic data
Age/Education level/Employment status/Marital status
B2. Origin of the situation
How are you related to the person that you look after? (mother/father, son/daughter, brother/sister, husband/wife…)
What illness/es does your relative suffer from? How does the illness affect their physical and/or mental capacities?
How long have you been looking after them? How did this situation come about?
B3. Information received
Have you received any information about how to take care of your relative? How were you given this information?
In the event of having to make decisions about healthcare, treatment, looking after your relative, who makes the decisions? How do you decide?
B4. Daily life, care experience and perception of your health
How many hours per day do you dedicate to providing care? What kind of care does your relative need?
What is a normal day like for you?
How do you think taking care of your relative affects your health? What about your emotional/psychological well‐being? What changes have you noticed in your emotional/psychological well‐being?
B5. Family, social and working life
How has taking care of your relative affected your social life?
How do you disconnect from your obligations?
How is your relationship with the rest of your family? Has this relationship changed since you started taking care of your relative? Do other members of your family help you?
How has taking care of your relative affected your working life?
B6. Final questions
How would you describe your experience as a caregiver?
Is there anything else you would like to add?

### 2.4 Data analysis

We analysed all the interview transcripts following thematic analysis as described by Braun and Clarke in their six‐step methodological guide.^22^ The data analysis was inductive, thus thematic construction was data‐driven; no initial hypothesis guided the preliminary coding and subsequent development of themes.

Three investigators performed an initial line‐by‐line coding of the interview transcripts, ensuring each interview had been coded by at least two of them independently to develop a robust and consistent code set. All the codes were then discussed and refined between the same three researchers. The resulting codes were then sorted into potential themes.

The elaborated themes were refined using the three stages proposed by Braun and Clarke for this part of the analysis, with the participation of all the authors. First, all the coded extracts for each theme were read thoroughly to check coherence in the pattern that led to that theme definition. Once necessary adjustments had been made, the preliminary themes were contrasted with the whole data set to refine them. Finally, a detailed analysis of each theme, including the meaning and scope, as well as its relationship with the other themes, was conducted and written based on the data extracts coded in each one.

## 3 RESULTS

Twelve people aged from 34 to 77 were interviewed (8 women and 4 men). Two of them had a declaration of total disability to work, and five were retired or unemployed. The majority of them (*n* = 8) were the intimate partner, and four participants were daughters of the CP patient (Table 2).

During data analysis, one overarching theme—‘Becoming a secondary actor of one's own life’—and three themes were elaborated: ‘Key elements that shape this experience’; ‘It's the hand that life dealt me’ and ‘The burden of being a caregiver’.

The overarching theme captures an idea that underpins the other three themes, while the combination of the themes ‘Key elements that shape this experience’ and ‘It's the hand that life dealt me’ lead to the third theme named ‘The burden of being a caregiver’.

### 3.1 Becoming a secondary actor of one's own life

This overarching theme is about how caring for their relative with CP, regardless of the relationship, was a central part of the lives of all the participants in the study, to the point of casting a shadow over their own lives and experiences. This shadow was expressed on the one hand by explicit statements about the full‐time dedication to the care, and by narratives about how they have minimized the importance of their own health issues and needs on behalf of those of their relatives, as the following quote exemplifies:

I have been raging with pain for 10 years, but as my husband was in pain I did not pay attention to mine and when I went to the doctor, he told me I no longer had a hip, it had completely worn out. I didn't pay attention to my pain because I was taking care of him. (Miriam, 66 years old, caregiver for her husband 24 hours per day)

On the other, this loss of relevance resulting from caring for the other person was reflected in the way participants answered the open questions. Although most of the questions were focused on their experiences as caregivers, all the interviews were full of detailed and rich information about the health issues, mental health status, fears, hopes, difficulties and so on of the persons they were taking care of, while theirs were described more succinctly or minimized (Table 3). In this same vein, they used ‘we’ to describe problems or issues that were in fact their relative's, as it is shown in the Ana quote (Table 3). Additionally, the IC has to defend the credibility of their relative in relation to the pain experienced against those who question the severity or even the existence of pain, as Natalia exemplified (Table 3).

**Table 3:** Quotations illustrating categories and theme.

Overarching theme: ‘Becoming a secondary actor of one's own life’
Natalia, 44 years old, caregiver for her husband: ‘I've already come to terms with what my daily life is like and it doesn't bother me and if it should bother me, I don't mind … because he's here for me and I'm here for him. I'm not going to put having no lymph nodes in my breast before saying to him, “give me your hand, I'll help you get up,” I don't mind. It's no trouble’.
Ana, 70 years old, caregiver for her husband: ‘I was recently admitted to the hospital here for my prostate. And now on the 25th we'll be hospitalized again. ‐ You will be hospitalized? ‐ Him, him. I'll be admitted with him. Who takes care of him? Me, so I have to be in hospital with him 24 hours a day’.
Ana, 70 years old, caregiver for her husband: ‘He had an operation on his knee and I always keep an eye on him and make sure he takes his medicine. His illness doesn't hinder me at all. The thing is there's just the two of us and it's normal, I cook for both of us, I go shopping for him, go to the chemist's, go to the doctor for him … Very often he doesn't even go to see the family doctor, I tell the doctor where it hurts, “Look, he's not getting any better, his leg hurts today” (as if speaking to the doctor)’.
Natalia, 44 years old, caregiver for her husband: ‘We found people who think you're faking it. Let's see, I'm telling you that this, this and this happens to him, they say: “it's that you … don't go every time with the crutches.” When you see him with the crutches, it's because he can't stand it more’.
Miriam 66 years old, caregiver for her husband: ‘If he calls me 20 times, I go 20 times … Having to look after him doesn't bother me at all, thank God. Tiredness is the only thing and when the night finishes you end up really tired because of the “bring me this, give me that” … you know. For his shower he sits in a chair and I shower him because he can't stay standing up … anyway’.
Marco 77 years old, caregiver for his wife: ‘I have my pains too. For example, I sometimes get sharp pains from my hip to my calf and I have to stop and squeeze my leg and that … I have problems with my neck and shoulders and I get dizzy, but no problem, I try to ignore it’.
Category: ‘Key elements that shape this experience’
Pablo, 74 years old, caregiver for his wife: ‘I am retired, I have a fairly decent pension. The thing is, I live in a rented house. So, half of my pension goes on rent, electricity, water … For example, this month I'm having problems making ends meet’.
Juan, 72 years old, caregiver for his wife: ‘Well, I could take her out for breakfast every day, for example, which she likes. We'd go by car, I'd take her to the shopping centre, take her into town to have a coffee, but I can't do that every day. I can't spend 4 or 5 euros every day. I do it from time to time’.
Marco, 77 years old, caregiver for his wife: ‘We requested the help of a professional caregiver from the Junta de Andalucía (regional government), and after a while they replied that I'm a Grade 1 dependency care case. We think Grade 1 is too little, so we appealed to be considered Grade 2. That was two years ago, it'll be two years soon, and still they haven't replied’.
Natalia, 44 years old, caregiver for her husband: ‘My husband is a builder. He can't work and he has a 55% disability. When the medical board called him and saw him, they said, “you can't work as a builder, but you can do other work”—“What other work? I can't stand up, I can't sit down and I can't spend a long time in the same position”’.
Rocío, 34 years old, caregiver for her husband: ‘The doctors advised my husband not to go up and down stairs and we live on the fourth floor with no lift. So, it's a nightmare every time he goes up or down the stairs’.
Pablo, 73 years old, caregiver for his wife: ‘My children only help if I'm a bit overwhelmed. I do tell them that they have to take their mother to certain places, but not very often, very rarely because it's not necessary if I'm alright … there's no need for me to give them work to do’.
Category: ‘It's the hand that life dealt me’
Milagros, 45 years old, caregiver for her mother: ‘I want her to be happy for the time she is with … while she is alive. And that she is as comfortable as possible, as happy as possible and that she feels loved and close, know what I mean? That's my aim, mothing else’.
Miriam 66 years old, caregiver for her husband: ‘I'm alright for now. I hope I can take care of him like this for a long time. And that God gives me good health. That's what I ask for. And that God gives him good health so I can take care of him for a long time. Yes. Because he's such a good man and he's been so good to me. Seeing him like this…’.
Marta, 47 years old, caregiver for her mother: ‘They are all my family. I like the mother hen and the others are my chicks. You know what I mean? So, I have never thought about living in any other way’.
Celia, 46 years old, caregiver for her mother: ‘My brother is not as competent as me, do you understand? I'm much better at these things. When my mother was in hospital for her hip, I was the one that help her have a shower, I was the one … you know? I'm more competent. For coming with her to the hospital, he's no use, and well I'm a bit better at it (she laughs)’.
Rocío, 34 years old, caregiver for her husband: ‘I have experience as a caregiver for my mother, who passed away 7 years ago, after a long cancer. So, at home we are experts at handling and managing situations that are a bit difficult, and carrying on with life despite illness. But it's purely down to experience and to the slaps in the face that life brings. Not because anyone has told us how to do it’.
Category: ‘The burden of being a caregiver’
Rocío, 34 years old, caregiver for her husband: ‘I much more tired than I should be. That's simple and obvious. My back hurts because I sometimes do more than I should. It affects me, of course it does. A lot. As far as care goes, there are no limits’.
Elena, 51 years old, caregiver for her mother: ‘If her illness gets more complicated in the future, we'll see what I do. Do you understand? So, now is when I start getting worried, but it's alright for now. Like I say, I want to be positive and anything I can do for her now … so that she gets better … well. But it affects me, of course it does, it's normal, isn't it?’
Celia, 46 years old, caregiver for her mother: ‘I haven't been out for a long time, and haven't wanted to either because I won't feel at ease knowing that my mother is alone’.
Natalia, 44 years old, caregiver for her husband: ‘Well, we hardly have a social life, we don't have a social life. I might see one of my friends, but we don't usually go out’.

### 3.2 Key elements that shape this experience

This theme englobes the codes that describe all the contextual elements beyond the individual characteristics of the caregiver and the person with CP that were essential in shaping the experiences as caregivers. The elements mentioned by the participants in this study included: the advanced age of both the caregiver and their patient, the caregiver's health status, the socioeconomic status, the COVID outbreak and the (mainly) lack of social/family support. In relation to age and the health status of the caregiver, some of the participants were themselves facing health issues related to ageing while simultaneously providing care for their relatives.

The relevance of economic status was repeatedly mentioned by participants in their discourse, especially when they were facing economic difficulties. Difficult financial circumstances were often related to a basic educational level and a life of unskilled work in precarious conditions. Living on a limited budget was described by interviewees as a source of worry and a limitation to the care they could provide, from the food they could buy to making the home accessible, and the possibility of employing someone to help them with the housework or the leisure activities they could plan. As Juan said: ‘My problems … the real problem I have is financial, more than her health. Well, her health is very important, but if we didn't have money problems, we might live much better’.

All the caregiver tasks were performed with no specific support from the healthcare services and with little support from social services, which were described as insufficient and slow to respond, as the Marco and Natalia quotes show (Table 3).

As this study was conducted in 2021, the COVID outbreak was mentioned by participants as a relevant event in their experiences. The most extreme impact was the loss of family members due to COVID, this being the case for two of the participants. In the remaining cases, the impact was related to the lockdown measures and the restrictions imposed by the government regarding leisure and public spaces, and whether they were afraid of going out due to the risk of infection. As Juan stated: ‘My wife isn't very inclined to go out and with this pandemic even less so; she's very scared’. In addition, the lockdown also had an impact on the physical and mental sphere, as Ana stated: ‘The lockdown has been absolutely awful for my husband because he has hardly moved for 3 months … but at least we had the treadmill’.

Yet the cornerstone of all these elements was the wider family/social support or the lack of it. The participants' experiences varied in this sense and ranged from very good emotional support to the feeling of being totally alone. In any case, the support received was strongly intertwined with all the elements described above, as the support the other family members could offer depended to a large extent on family members' age, socioeconomic status or employment status. Thus, in some of the narratives, participants explained or justified the lack of support from their relatives by explaining the economic or health struggles those relatives were dealing with.

My husband's sisters' have settled lives, so they don't have much contact. If we won the lottery, they'd all come round, but nobody comes to deal with the pain. (Natalia, 44 years old, caregiver to her husband)

### 3.3 It's the hand that life dealt me

Although it was not a question included in the interview guide, participants explained or somehow justified why they were in that role. The diverse reasons given were underpinned to different extents by statements about having no other option but to remain in the role with a sense of resignation. This lack of alternatives was strongly linked to the previous category, where many participants described the lack of wider support. All these different positions were sustained by feelings of responsibility and obligation because of love or in the case of daughters with parents, the feeling of having to repay the care their parents had given them, as Milagros explained:

I took on the responsibility myself because, in truth, it's what my parents have always done for me. I'm the eldest, I was given the responsibility and that's how it has stayed. I've never wanted to stop … not because I wouldn't like to but because I'd feel bad, like selfish, if I did, you know what I mean? (Milagros, 45 years old, caregiver to her mother)

Their caregiver role implied a long list of tasks. Participants described how they were in charge of medical appointments, dealing with medication, managing medical information, making decisions, keeping their relative company, doing the housework, trying to cheer up their relatives or even assuming the cost of moving house, while at the same time having to cope with their own worries and anxiety about the future.

Well, you see, my husband needs me to put his socks on because he can't due to the pain. I have to shower him … he walks with the walking frame, with a walking stick, but when I come to the hospital I bring him with the wheelchair because he can't walk for more than 10 minutes. So, except for feeding him, I have to do nearly everything for him. (Miriam, 66 years old, caregiver to her husband)

In the description of what taking care involved, it is of note how the narratives of the women differed from those of the men. When describing the activities, they did as part of the care, men mentioned explicitly doing the housework while women did not. Moreover, men referred to the tasks they did as providing ‘help’ to their partner and not one of their responsibilities in the house. As Juan said:

I help her, she practically … I even help her make the lunch, something I didn't know how to do before, but there's no alternative but to learn. I clean for her, make the beds for her, go shopping for her…. (Juan 72 years old, caregiver to his wife)

In some cases, they described how despite living with CP their wives still did most of the housework. Pablo stated that: ‘my wife is a housewife and, no matter how much I want to, she says she prefers to do the cleaning herself. She does everything to be honest; she's a traditional woman and that's alright. She doesn't stop from the moment she gets up. Our daughters have left home so the only responsibility she has, you could say, is me’.

In this sense, women participants felt better equipped to assume the caregiver role as most of them had previous experience of taking care of someone else (Table 3), while for the men who took part in this study, this was their first experience in this role.

I've always been someone that … I've taken care of my father, I was with my brother when he was ill, when my sister had a breast removed too … So, I'm someone that can adapt to any situation and it doesn't affect me, thank God. I mean, losing a loved one affects me. That gets you down, but then you say ‘Well, if that's the way it is…’ If God meant it to be like this … it's the hand that life dealt me. (Miriam, 66 years old, caregiver to her husband)

In fact, for some women participants taking care of their relative with CP was simultaneous to taking care of their children or grandchildren, despite the extra responsibility and time it implies. As Elena, who takes care of her mother, said:

I'm also at an age where, it's not that I'm very old, but you know, I do the housework, take the girls to school, come back to look after my mother; I go with her, up, down, running around … stress, lots of stress. And then my back and neck feel the effects and it affects me. I have contractures, I get dizzy and I have to take Enantium and Diazepam all the time. It affects me a lot. (Elena, 51 years old, caregiver to her mother)

### 3.4 The burden of being a caregiver and coping strategies

The combination of the different components described in the previous two themes leads to a wide range of perceived burdens resulting from the caregiver's role. The narratives of those participants who were wealthier and had good family or social support expressed fewer consequences for their physical and mental health than other participants who were struggling with other issues such as limited economic resources.

However, it is important to note that under this theme and within each interview there were inconsistencies, in the sense of participants saying there were no consequences and later describing some, such as a very limited social life, abandoning hobbies to take care of their relative, physical issues, anxiety, fear for the future, emotional exhaustion or physical consequences to cite some examples (Table 3).

I take pills and I have been to many psychologists and they all say the same: ‘it's your mother … You are your mother's mother and, in the end, that muddles everything’. (Marta, 47 years old, caregiver to her mother)

At the same time, statements showing resilience, hope and a positive attitude were commonplace. In line with them, participants described their coping strategies and how they were making an effort to have their own space or, in some cases, after realizing they were somehow losing their life, making a conscious effort to regain it.

Uff … if you don't want to bang your head against the wall along the way, it isn't a physical journey, it's a mental one. It's mental. How you are influenced … by certain emotions and you don't understand … you have to understand that, despite the pain, life goes on. (Rocío, 34 years old, caregiver for her husband)

## 4 DISCUSSION

The findings of this study show that being an IC for a relative with CP became the most important role in the IC's life, to the point of casting a shadow over the priorities of their own lives. The experiences also varied depending on the contexts and key elements such as socioeconomic level or family support. Likewise, independently of the context, the ICs had the feeling of being the only person responsible and able to perform the care, regardless of the consequences.

A significant result of this study was that the ICs interviewed neglected to live their own lives to care for their relative with CP. It is remarkable how the ICs referred to their relative's illness as if it were their own and to the pain process as a shared experience. These findings are in line with studies conducted to assess the needs of ICs of individuals with other pathologies, where participants lacked time to care for themselves and address their own health concerns,^23^ in some cases with severe detrimental consequences for their health. It has been shown how CP becomes the focus of patients' lives, and they have to both redefine their identity and adjust to the new constraints and physical limitations of their bodies.^24^ In this line, and in the context of our findings, ICs change their daily lives according to treatment regimes, helping to manage pain and side effects, attending medical appointments and resolving everyday problems.^9^, ^25^ Thus, the length of time spent on care is not the only important predictor of the overall burden on ICs.^8^, ^26^ There is also the time lost for themselves, the lack of privacy and the development of a new identity as a caregiver.^27^ The enormous impact the CP had on the lives of the IC found in this study suggests the concept of ‘we‐disease’ can be applied to the described experiences. This concept, developed initially by the participants of a study about stress and coping strategies among breast cancer patients and their partners, reflects how breast cancer impacts not only the patient but their intimate partners as well and, furthermore, how the coping strategies with the stress of each of the members of the couple are interrelated.^28^, ^29^ Future research on the experiences of IC could further explore the applicability of the ‘we‐disease’ concept to IC and enrich knowledge about their experiences by adding this interrelational dimension.

Social and structural determinants of health related to the burden of people suffering from CP have been widely studied.^30^, ^31^ Our study enhances this knowledge by highlighting how social determinants of health are also a key element in the burden of being an IC. The limited budget was described by interviewees as a source of worry and a limitation to the care they could provide, and thus contributed to the perceived burden. Moreover, economic difficulties aggravated the decrease in the time that both the IC and the CP patient had for leisure and social activities. This finding is relevant in light of the results from Miller et al.,^11^ who found the inability or difficulty to pay for basic needs and not having a social life were related to a high prevalence of depression among ICs. It has been argued that no other health problem causes as much disability as CP.^32^ In this line, the lack of economic resources to make the patient's home accessible prevented both the patient and their family member from spending more time outside of their home. Prior studies^33^ have shown housing as an important factor driving health inequalities, with long‐term isolation producing adverse effects on mental well‐being. Various studies suggest that among the variables that have contributed to the current crisis in CP care are policies that influence the socioeconomic climate of the healthcare system.^34^ In this line, our results indicate ICs experience a feeling of helplessness due to a lack of resources provided by public health or social services, such as financial support or formal caregivers. Therefore, addressing social inequalities associated with CP is an essential initial step in improving this health problem, using collaborative approaches based on the chronic care model, which would optimize not only the patients' quality of life but also reduce the burden on caregivers.^35^

This study was conducted in the context of the COVID‐19 pandemic. The fear of contracting COVID‐19, along with public health measures such as home confinement, increased exponentially the time spent at home. Recent literature^36^ has emphasized that one of the most important consequences of the lockdown was its impact on mental health, particularly fear, anxiety and negative thoughts about oneself and the future.^37^, ^38^ In this respect, the lockdown together with a combination of the different factors resulting in a limited social life—taking care of their relative, physical issues, emotional exhaustion or physical consequences—lead to an increase in the burden perceived by the caregiver. Future public health measures like those implemented during this pandemic should take into consideration the impact they have on vulnerable populations to minimize health inequities instead of increasing them.

However, the caregiver burden is not a universal experience.^27^ Some individuals are able to adapt easily to the responsibility and demands of caregiving, whereas others report significant strain and distress.^39^ In line with previous research, the study showed intergroup differences in gender, depending on the relationship with the family, sources of support, duration of care and stage of the disease. The findings suggest that gender and the type of relationship are important concepts in understanding the caregiving process and that they are often interwoven. In the case of the women, there were differences in recognizing them as a caregiver since they assume the role motivated by love and the desire to return the love received by their relatives. In fact, some of them had played or were playing this role with various members of their families simultaneously, accepting that they had no choice. This finding is in consonance with previous research on the ‘sandwich generation’.^19^ Men and women have been shown to cope differently with caregiving situations.^40^ Women are more concerned with the enhancement of others' emotional well‐being and with the provision of emotional support. They are more emotionally involved in the caregiving, while at the same time being largely responsible for doing the housework. Men have a more task‐oriented approach to caregiving. This suggests that both the level and impact of the burden develop differently over time for men and women, as other articles have shown.^41^

## 5 STRENGTHS AND LIMITATIONS

As previously described, several steps were taken to strengthen the trustworthiness of the findings. They do, however, need to be interpreted with some limitations in mind. Concerning transferability, it is important to consider the context in which this study was conducted: a group of individuals who accompanied their relatives to a pain clinic in the Spanish healthcare system. With this in mind, the results from this study could be relevant for understanding the experiences of ICs for a relative diagnosed with CP in other countries with a similar sociocultural background and healthcare systems since the consequences they face and concerns they have may be similar.

Regarding credibility, participants with different sex/gender, ages and experiences were chosen to increase the likelihood of shedding light on the research question. However, the vast majority of people interviewed were women. Nonetheless, as discussed in this study, this is in line with the percentage of women who tend to be caregivers since they assume the role motivated by love, while also accepting that they have no choice.

Another limitation of this study is that, although to take part in this study the participants had to consider themselves to be the main caregiver, the results suggested gender differences in the way the idea of care is conceived and understood. However, the data were not rich enough to support a deep analysis and the elaboration of conclusions about the social construction of the term. Further research with this aim is required.

## 6 CONCLUSION

This study's findings highlight how the CP impacts IC lives. Being an IC for a relative with CP became the most important role in the IC's life, to the point of casting a shadow over their own needs. Besides, participants felt not having other options but to keep going with that role. Yet, the context was essential in shaping the experiences as caregivers and the burden derived from caregiving. In this line, differences related to gender roles were found in the narratives of participant women and men.

### 6.1 Practice implication

The number of people suffering from CP is expected to continue rising, and consequently so will the number of IC. This study shows that ICs of individuals with CP have specific needs similar to the IC of individuals with other chronic conditions but with certain specificities. The lack of a formal caregiver provided by the state and delays in financial help lead to the family member feeling more and more isolated with greater responsibility and a bigger burden, leading to physical and mental problems. This should be taken into account in the implementation of policies and healthcare programs aimed at the attention of individuals living with CP.

Finally, IC should be considered an integrated part of the CP illness process by healthcare providers. In this enhanced person‐centred care, to meet both patient and IC's needs, social determinants of health and social support should be assessed in each individual case from a gender perspective to implement evidence‐based measures that prevent negative consequences for IC.

## CONFLICT OF INTEREST

The authors declare no conflict of interest.

## ETHICS STATEMENT

The study protocol was approved by the Clinical Research Ethics Committee (Reference Number of the study: EXPCUI2020), ensuring compliance with the standards of good clinical practice. All informants gave their consent to participate after they had received individualized and sufficient information. This also included the possibility of taking back the consent to participate.

## Data Availability

The data that support the findings of this study are available on request from the corresponding author. The data are not publicly available due to privacy or ethical restrictions.
